# The Microcephaly Epidemic Research Group Paediatric Cohort (MERG–PC): A Cohort Profile

**DOI:** 10.3390/v13040602

**Published:** 2021-04-01

**Authors:** Demócrito de Barros Miranda-Filho, Elizabeth B. Brickley, Anna Ramond, Celina Maria Turchi Martelli, Nuria Sanchez Clemente, Thália Velho Barreto de Araújo, Laura Cunha Rodrigues, Ulisses Ramos Montarroyos, Wayner Vieira de Souza, Maria de Fátima P. M. de Albuquerque, Liana O. Ventura, Ernesto T. A. Marques, Mariana C. Leal, Sophie H. Eickmann, Maria Angela Wanderley Rocha, Paula Fabiana Sobral da Silva, Maria Durce Costa Gomes Carvalho, Regina Coeli F. Ramos, Danielle Maria da Silva Oliveira, Morgana do Nascimento Xavier, Rômulo A. L. Vasconcelos, Andreia Veras Gonçalves, Alessandra Mertens Brainer, Marli Tenório Cordeiro, Ricardo Arraes de Alencar Ximenes, on behalf of the Microcephaly Epidemic Research Group

**Affiliations:** 1 Pós-Graduação em Ciências da Saúde, Universidade de Pernambuco, Recife 50100-010, Brazil; ulisses.montarroyos@upe.br (U.R.M.); paulafsobral@yahoo.com.br (P.F.S.d.S.); mdurce2@gmail.com (M.D.C.G.C.); dllsilva@gmail.com (D.M.d.S.O.); romulo.alv@hotmail.com (R.A.L.V.); brainerlima@aol.com (A.M.B.); raaximenes@uol.com.br (R.A.d.A.X.); 2 Department of Infectious Disease Epidemiology, London School of Hygiene & Tropical Medicine, London WC1E 7HT, UK; elizabeth.brickley@lshtm.ac.uk (E.B.B.); anna.ramond86@gmail.com (A.R.); nuriasanchezclemente@gmail.com (N.S.C.); laura.rodrigues@lshtm.ac.uk (L.C.R.); 3 Instituto Aggeu Magalhães—Fundação Oswaldo Cruz (Fiocruz), Recife 50740-465, Brazil; turchicm@gmail.com (C.M.T.M.); waynervieira@gmail.com (W.V.d.S.); militaofatima@gmail.com (M.d.F.P.M.d.A.); emarques@cpqam.fiocruz.br (E.T.A.M.); morganasci_xavier@yahoo.com.br (M.d.N.X.); marli.tenorio@gmail.com (M.T.C.); 4 Departamento de Medicina Social, Universidade Federal de Pernambuco, Recife 50670-420, Brazil; thalia.araujo@ufpe.br; 5 Departamento de Oftalmologia, Fundação Altino Ventura, Recife 52171-011, Brazil; lianaventuramd@gmail.com; 6 Departamento de Cirurgia, Universidade Federal de Pernambuco, Recife 50670-420, Brazil; marianacleal@hotmail.com; 7 Departamento Materno-Infantil, Universidade Federal de Pernambuco, Recife 50670-420, Brazil; sophie.eickmann@gmail.com; 8 Hospital Universitário Oswaldo Cruz, Recife 50100-130, Brazil; mangelarocha@uol.com.br (M.A.W.R.); coeliramos@hotmail.com (R.C.F.R.); andreiaverasg@gmail.com (A.V.G.); 9 Pós-Graduação em Medicina Tropical, Universidade Federal de Pernambuco, Recife 50670-901, Brazil

**Keywords:** congenital Zika syndrome, microcephaly, paediatric cohort

## Abstract

This cohort profile aims to describe the ongoing follow-up of children in the Microcephaly Epidemic Research Group Paediatric Cohort (MERG–PC). The profile details the context and aims of the study, study population, methodology including assessments, and key results and publications to date. The children that make up MERG–PC were born in Recife or within 120 km of the city, in Pernambuco/Brazil, the epicentre of the microcephaly epidemic. MERG–PC includes children from four groups recruited at different stages of the ZIKV microcephaly epidemic in Pernambuco, i.e., the Outpatient Group (OG/*n* = 195), the Microcephaly Case–Control Study (MCCS/*n* = 80), the MERG Pregnant Women Cohort (MERG-PWC/*n* = 336), and the Control Group (CG/*n* = 100). We developed a comprehensive array of clinical, laboratory, and imaging assessments that were undertaken by a ‘task force’ of clinical specialists in a single day at 3, 6, 12, 18 months of age, and annually from 24 months. Children from MCCS and CG had their baseline assessment at birth and children from the other groups, at the first evaluation by the task force. The baseline cohort includes 711 children born between February 2015 and February 2019. Children’s characteristics at baseline, excluding CG, were as follows: 32.6% (184/565) had microcephaly, 47% (263/559) had at least one physical abnormality, 29.5% (160/543) had at least one neurological abnormality, and 46.2% (257/556) had at least one ophthalmological abnormality. This ongoing cohort has contributed to the understanding of the congenital Zika syndrome (CZS) spectrum. The cohort has provided descriptions of paediatric neurodevelopment and early epilepsy, including EEG patterns and treatment response, and information on the frequency and characteristics of oropharyngeal dysphagia; cryptorchidism and its surgical findings; endocrine dysfunction; and adenoid hypertrophy in children with Zika-related microcephaly. The study protocols and questionnaires were shared across Brazilian states to enable harmonization across the different studies investigating microcephaly and CZS, providing the opportunity for the Zika Brazilian Cohorts Consortium to be formed, uniting all the ZIKV clinical cohorts in Brazil.

## 1. Introduction

The Microcephaly Epidemic Research Group Paediatric Cohort (MERG–PC) is a geographically defined longitudinal cohort of children with confirmed or suspected prenatal exposure to Zika virus (ZIKV), born during the 2015–2017 ZIKV epidemic in Pernambuco State, Brazil. The cohort was set up in response to the increase in cases of microcephaly reported initially in northeastern Brazil and subsequently across the country.

The cluster of microcephaly cases reported between August and October 2015 led the Brazilian Ministry of Health to declare a Public Health Emergency of National Importance in November 2015 [1]. At the time of the declaration, the state of Pernambuco had reported the highest number of suspected cases of microcephaly (646 cases), followed by Paraíba (248 cases) and Rio Grande do Norte (79 cases) [2].

**Figure 1:**
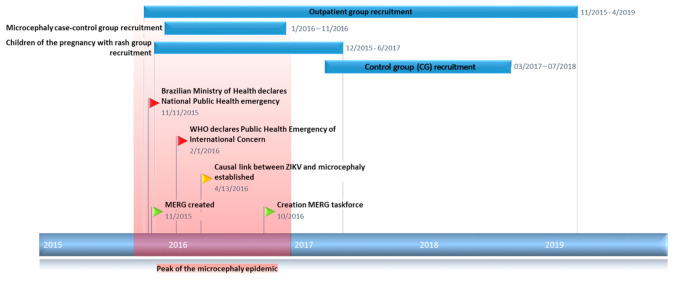
Study timeline.

The Microcephaly Epidemic Research Group (MERG) [3] was founded in November 2015 by a multidisciplinary and multi-institutional team of researchers, with the support of the Brazilian Ministry of Health and the State Health Secretariat of Pernambuco. Despite financial constraints, and in the midst of the unfolding public health emergency, the MERG initiated the first large-scale case–control study [4,5] investigating the hypothesis linking prenatal ZIKV infection and microcephaly (Figure 1).

As further Brazilian and international studies corroborated the findings of the case–control study and the link between microcephaly and prenatal ZIKV exposure became accepted by the scientific community, the MERG initiated a series of studies to investigate the full spectrum of clinical consequences associated with in utero ZIKV exposure. MERG–PC study population includes children recruited from four MERG studies at different stages of the ZIKV microcephaly epidemic in Pernambuco.

This cohort profile aims to describe the ongoing follow-up of children in the MERG–PC. The profile details the context and specific aims of the study, the study population, the methodology including clinical assessments and diagnostic testing, and key results and publications to date.

## 2. Aims and Objectives

The overall aim of the MERG–PC is to describe the clinical, anthropometric, and neurodevelopmental spectrum of congenital Zika syndrome (CZS) over the early life course.

Specific study objectives include the following:

- To characterise the clinical spectrum of CZS in the first four years of life;
- To describe the children’s growth trajectories, including weights, heights, and head circumferences, in the first four years of life;
- To detail the children’s attainment of neurodevelopmental milestones in the first four years of life;
- To quantify the children’s morbidity and mortality in the first four years of life.

Children were classified according to their exposure status and clinical characteristics as follows:

(i) Presumably exposed children with congenital microcephaly born during the period of the microcephaly epidemic, but without laboratory confirmation of maternal ZIKV infection during pregnancy;
(ii) Presumably exposed children with other potentially Zika-related abnormalities born during the period of the microcephaly epidemic but without laboratory confirmation of maternal ZIKV infection during pregnancy;
(iii) Confirmed or presumably exposed children with or without apparent congenital abnormalities born during the period of the microcephaly epidemic with laboratory evidence of maternal ZIKV infection during pregnancy;
(iv) Unexposed children born during the period of the microcephaly epidemic with clinical and laboratory evidence against maternal ZIKV infection during pregnancy or without laboratory evidence of ZIKV infection.

## 3. Study Site

The children that make up the MERG–PC were born in the Recife metropolitan region, or within 120 km of the city, in Pernambuco, Brazil.

The population of Pernambuco state is 9.4 million, of which almost 4 million live in the Recife metropolitan region [6]. The climate in the region is tropical savannah according to the Köppen–Geiger classification with a mean annual temperature of 25.8 °C and mean total annual rainfall of 1804 mm [7]. In the municipality of Recife, the infant mortality rate is 10.8 deaths per 1000 live births, 97% of 6–14-year-olds attend school, and 69% of the population reside in households with a sanitary sewage system [8].

## 4. Study Population/Recruitment

The northeast of Brazil was the epicentre of the microcephaly epidemic, and all investigations had to be designed, planned, and conducted when it was already occurring. MERG adopted several strategies for identifying participants and coordinating research as part of the rapid response to the epidemic.

First, MERG developed formal partnerships with the tertiary care teams at reference hospitals for children affected by CZS. The research partnerships included collaborations with specialists in paediatric infectious disease, paediatric neurology, otorhinolaryngology, ophthalmology, paediatric urology, paediatric endocrinology, imaging, speech therapy, and neurodevelopment who had been providing specialist input to the care and study of these children since the onset of the microcephaly epidemic. This coordination with clinical care teams enabled, after ethical approval (number of approval CAAE: 52803316.8.0000.5192, Comitê de Ética do Complexo Hospitalar HUOC/PROCAPE), standardised data collection on research forms during outpatient visits, even before funding resources were available for research activities in the field.

Second, families who participated in the 2016 MERG-led case–control study of ZIKV and microcephaly were invited to participate in longer-term follow-up in the MERG–PC after the field team was formed. All had a baseline assessment at birth, and the resulting information was integrated into the MERG–PC database.

Third, MERG worked with the Pernambuco State Health Secretariat to coordinate protocols for public health surveillance and research efforts. The resultant epidemiological surveillance program (Center for Strategic Information on Health Surveillance in Pernambuco; Cievs/PE) received compulsory notifications of pregnant women with rash. The women registered in the notification system were invited to participate in the MERG Pregnant Women Cohort (MERG–PWC) and the children of participants in the MERG–PWC were recruited to join the MERG–PC.

Fourth, a control group of 100 ZIKV-negative children (by real-time reverse-transcription polymerase chain reaction (qRT-PCR)) at birth and born to women who repeatedly tested negative for ZIKV (by qRT-PCR and immunoglobulin (Ig) M) during pregnancy, from the Pernambuco centre that participated in the International Prospective Observational Cohort Study of Zika in Infants and Pregnancy (ZIP Study). This group makes up the most thoroughly tested comparator sample of unexposed children born in the period of the ZIKV and microcephaly epidemics in Pernambuco state.

The MERG–PC thus includes children from four different groups recruited at different stages of the ZIKV microcephaly epidemic in Pernambuco (see Table 1). This recruitment strategy was chosen to maximise the total number of individuals in the study population.

The children classified according to their exposure status and clinical characteristics (see Section 2. Aims and Objectives) were grouped according to the source of the recruitment and referred to as the Outpatient Group (OG) (composed of children classified as i and ii), the Microcephaly Case–Control Study (MCCS) (composed by children classified as i and iv) [4,5], the MERG Pregnant Women with Rash Cohort (MERG–PWC) (composed by children classified as iii and iv) [9] and the Control Group (CG) (composed by children classified as iv) [10]. The recruitment for each group is described below.

### 4.1. Outpatient Group (OG)—Recruitment 11/2015–04/2019

Recruitment into MERG–PC began with the OG in November 2015. Children with a head circumference below 33 cm (i.e., the initial Brazilian Ministry of Health microcephaly definition) and/or with severe central nervous system (CNS) malformations as determined by postnatal computerised tomography (CT) findings who were referred to a specialist centre (Hospital Universitário Oswaldo Cruz) in Pernambuco were invited to participate in the MERG–PC study. Shortly after recruitment for the OG began, the definition of Zika-related microcephaly was revised through scientific community consensus [11,12] to a head circumference z-score <−2 (equivalent to two standard deviations below the mean for gestational age and sex). In March 2016, the Brazilian Ministry of Health adopted this threshold, and MERG updated recruitment accordingly [13]. As recruitment for the OG began during the peak of the epidemic, this group includes a high number of children with a severe CZS phenotype. Of note, some children born before November 2015 who presented characteristic CZS phenotypic features were also recruited. This is because some caregivers presented to the hospital after hearing about this new congenital infection and recognizing the phenotypic features in their children.

### 4.2. Microcephaly Case–Control Study (MCCS)—Recruitment 01/2016–11/2016

The second source of recruitment began in January 2016 with the MCCS. The aim of this case–control study was to provide robust evidence to support or refute the working hypothesis that in utero ZIKV exposure was associated with congenital microcephaly. Details of the study and findings have been published previously [4,5].

Neonates born to women residing in Pernambuco State and delivered in one of eight public maternity units in the metropolitan region of Recife were eligible to be enrolled in the study. Cases were neonates with microcephaly, defined as a head circumference of at least two standard deviations below the mean for sex and gestational age according to the Fenton growth chart [14]. Controls were live neonates born without microcephaly or major congenital malformations as confirmed by a normal physical examination and normal cranial ultrasound carried out by a neonatologist at birth. For each case, two controls were selected from the first neonates born the following morning in one of the study hospitals, matched by administrative health region of residence and expected date of delivery to ensure cases and controls were conceived at the same stage of the epidemic. Both cases and controls were invited to join MERG–PC. Of note, head circumferences z-scores were subsequently recalculated according to the INTERGROWTH-21st charts [15,16] since this became the standardised tool of choice among ZIKV cohorts.

### 4.3. MERG Pregnant Women Cohort (MERG–PWC)—Recruitment 12/2015–06/2017

In December 2015, the Pernambuco State Health Department introduced a surveillance system for pregnant women presenting with rash (Cievs/PE, cievspe.com) [9]. Once notified to Cievs/PE, and ideally, within five days of rash onset, officials from the State Health Secretariat registered women and collected a first blood sample for ZIKV testing.

For the MERG–PWC, the MERG invited pregnant women registered in Cievs/PE, and any other pregnant women presenting to local health services with rash, to join the pregnancy cohort, and their children to join the MERG–PC after birth. Initial recruitment was limited to the metropolitan region of Recife. To increase the sample size of women with laboratory evidence of ZIKV infection and the precision of the estimates of the risk of adverse outcomes in children, in April 2016, the catchment area for recruitment was expanded beyond Recife to include any pregnant women with rash and laboratory evidence of ZIKV infection residing within approximately 120 km of the city. No exclusion criteria were applied.

More than half of the notifications were received from six hospitals: Hospital João Murilo and Policlínica de Vitória, Instituto de Medicina Integral Prof. Fernando Figueira, Centro Integrado de Sáude Amauri de Medeiros, Hospital Agamenon Magalhães, Hospital Barão de Lucena, and Maternidade Bandeira Filho. MERG-associated fieldworkers collected a second blood sample from the pregnant women (i.e., at least 14 days following initial notification) and administered a detailed questionnaire. Overall, 503 pregnant women in the cohort were successfully followed until the end of pregnancy.

### 4.4. Control Group (CG)—Recruitment 03/17–07/2018

This group is composed of children born to women recruited by the ZIP Study who repeatedly tested negative for ZIKV during pregnancy and were followed up to the end of the first year of life by the MERG ZIP team and subsequently enrolled in MERG–PC and followed by MERG. The pregnant women were recruited in the first and early second trimesters, regardless of the presence of symptoms of ZIKV infection. Recruitment took place at 36 health units that offer prenatal care in the city of Recife. The women were followed by the research team until delivery. A detailed description of the ZIP Study Protocol was published elsewhere [10].

## 5. Baseline Assessments

In order to capture as broad a presentation of CZS as possible, MERG developed a comprehensive clinical assessment program, which is described below (Table 2). However, given the financial and logistical constraints posed by conducting a large-scale outbreak investigation during a live public health emergency, MERG also developed a strategic approach to allocating resources. Assessments were prioritised for children with a diagnosis of microcephaly and those with a higher likelihood of in utero exposure to ZIKV (i.e., children of lab-confirmed cases with maternal ZIKV infection in the MERG–PWC). Further, the age at which baseline assessments were conducted in the children varied as recruitment into the MERG–PC happened at different ages due to the multi-faceted recruitment strategy.

While a centralised assessment centre was being established at the Fundação Altino Ventura, and in order to avoid delays in clinical evaluation, clinical assessment of children recruited prior to October 2016 was performed locally by the same clinicians using the same questionnaires, allowing for minimal variation. Assessments were conducted at the University Hospital (Oswaldo Cruz) for children of the OG, at the maternity ward where the child was recruited for the MCCS children, and at Barão de Lucena for the children of the MERG–PWC. Since October 2016, all assessments of the children in the cohort have been performed at the Fundação Altino Ventura. During study visits, children complete a comprehensive program of tests and evaluations by a ‘task force’ of clinical specialists in a single day. Transportation to and from the assessment centre is provided for the caregivers and children.

Baseline evaluation of the children of CG was performed at the maternity hospitals at birth and included clinical and laboratory assessments (see detail in the ZIP Study Protocol) [10].

## 6. Clinical Assessments

Children in all four groups of the cohort were invited to participate in a comprehensive series of baseline and follow-up clinical assessments, according to the schedule presented in Table 2 and Table 3. These included neonatal examination and collection of delivery-related data for children recruited at birth, evaluation by a paediatric neurologist, collection of anthropometrics (i.e., head circumference, length, and weight), ophthalmological review with specialised tests including RetCam (Natus Medical Inc., Pleasanton, CA, USA), eye structural evaluations and visual acuity testing, and otorhinolaryngological clinical evaluations and auditory brainstem response (ABR) testing.

Neuroimaging evaluations reflect the age of the children at recruitment and clinical presentation. Additional neuro-imaging assessments are performed whenever medically indicated. For children without microcephaly in MERG–PWC group, cranial (transfontanellar) ultrasound was performed in the first six months of life to investigate brain calcifications and assess the degree of CNS damage. For all children with presumed or confirmed ZIKV exposure, computed tomography (CT) scans were performed in children with microcephaly or other sequelae at any time as clinically indicated. For children older than one month at first assessment, hospital records of CT scans performed at birth were reviewed as available. For children without microcephaly for whom cranial ultrasound results in the first six months of life were not available, an electroencephalogram (EEG) was performed to assess baseline brain electrical activity and investigate the presence of epileptogenic foci. For children with microcephaly or other neurological signs or symptoms, EEG assessments were performed approximately every six months or any time as clinically indicated. In addition, children with microcephaly underwent magnetic resonance imaging (MRI) of the brain at the end of the second year of follow-up, in order to make a final CNS assessment at an age where the normal myelination process is complete in children.

During study visits with the clinical task force, children were screened for neurodevelopment and behaviour, using instruments validated for use in Brazil. Specifically, assessment using the Survey of Well-Being of Young Children (SWYC) test [17] was performed at the three-month assessment or the first study visit if the child was older than three months at the time of enrolment. Thereafter, SWYC screening was repeated at every study visit (i.e., every 6 months until 2-years of age and every 12 months thereafter). For children without microcephaly, comprehensive neurodevelopmental assessments were performed using the Bayley Scales of Infant and Toddler Development^®^|Third Edition (Bayley^®^-III) in two specialised labs at the University Hospital (Oswaldo Cruz).

Children were assessed for oropharyngeal dysphagia, at baseline, and/or at least one time point during the follow-up, using clinical swallowing assessments of the stomatognathic system and a questionnaire administered to caregivers [18].

Subgroups of children with Zika-related microcephaly underwent further evaluations to investigate adverse outcomes and complications suspected to be related to neurologic damage. For example, those with moderate or severe oropharyngeal dysphagia were referred to further evaluation with video fluoroscopy or video endoscopy. Similarly, subgroups were evaluated by paediatric urologists to investigate neurogenic bladder and other abnormalities in the urogenital tract and by endocrinologists to investigate hormonal dysfunction.

Children of the CG also underwent anthropometric, clinical ophthalmological, and hearing assessment in the task force at the Fundação Altino Ventura. Neurodevelopment was assessed with the Ages and Stages Questionnaire (ASQ) and the Bayley^®^-III, according to the ZIP protocol [10].

### 6.1. Assessment of Microcephaly

An assessment of microcephaly was performed by a MERG-affiliated paediatrician when possible. Otherwise, head circumference at birth along with gestational age and sex were retrieved from medical records when available. At the first MERG visit, the presence of microcephaly was reassessed by MERG paediatricians. Microcephaly, for the purposes of the MERG–PC, is defined as a head circumference z-score of at least two standard deviations below the mean for sex and gestational age. The INTERGROWTH curves [15] were used. Of note, however, the microcephaly cases in the MCCS group were recruited prior to WHO having issued recommendations with regard to the definition of microcephaly, and, therefore, for the MCCS group, microcephaly cases were initially defined as a head circumference at birth of at least two standard deviations below the mean on the Fenton growth chart [14]. For standardisation, these children were later reassessed based on the INTERGROWTH curves [15,16].

### 6.2. Diagnostic Testing

ZIKV status was determined for the children of MERG–PC in different ways, according to their recruitment group (see Table 2).

For the OG, at recruitment, blood samples were collected from the new-borns and their mothers, and serum samples were tested for the presence of ZIKV (viral RNA and/or specific IgM), and TORCH agents (IgM and IgG for toxoplasmosis, rubella, cytomegalovirus (CMV), herpes simplex virus, syphilis) in order to rule out co-infections and/or to exclude other potential infectious causes of congenital microcephaly.

For the MCCS group, laboratory testing was performed in all study participants after delivery by MERG research staff. Serum samples were obtained from mothers and neonates and cerebrospinal fluid (CSF) was obtained from cases with microcephaly. As previously described [4,5], samples were tested for ZIKV by qRT-PCR and capture-IgM enzyme-linked immunosorbent assay (MAC ELISA). The presence of Zika virus and dengue virus (1–4)-specific neutralising antibodies was assessed in the serum samples of mothers and neonates (cases and controls) by the plaque reduction neutralisation test (PRNT_50_), with a 50% cut-off value for positivity. Serum samples were also tested for toxoplasmosis, rubella, and CMV, the other main infectious causes of microcephaly. Neonatal umbilical cord blood was also tested by qRT-PCR for ZIKV, chikungunya (CHIKV), dengue viruses (DENV) and toxoplasmosis, and CSF for ZIKV, CHIKV, and DENV.

Laboratory testing for ZIKV (by a combination of qRT-PCR, IgM, IgG3, and/or plaque reduction neutralisation test (PRNT_50_)) was also performed in sera for the majority of mothers in the MERG–PWC [9]. First blood samples were collected ideally within five days of rash by the State Health Secretariat the women were referred to or by MERG staff. Second blood samples were collected at least 14 days after notification of rash and a third and final sample was collected after delivery. Subsamples of maternal sera underwent testing for other potential infections, including CHIKV, DENV, toxoplasmosis, CMV (by qRT-PCR, IgG, and/or IgM), and rubella (by IgG and/or IgM).

Any historical diagnostic testing results were retrieved, when possible, from maternal and new-born medical records or the Cievs/PE notification system.

Maternal participants of the CG were tested for anti-ZIKV IgM antibodies, in parallel to DENV IgM, using the serological assay MAC ELISA (CDC protocol) and for ZIKV-RNA (qRT-PCR). Assays to detect antibodies to toxoplasma, rubella, CMV, HSV, and test for syphilis were performed with the equipment ‘ANALISADOR ELECSYS’ (model COBAS e 411), Hitachi High-Technologies Corporation—Roche Diagnostics GMBH, Germany. A serological assay was performed for the detection of anti-CHIKV IgM antibodies (Euroimmun, Lubeck, Germany). Infants were tested at birth for anti-ZIKV IgM and ZIKV-RNA, and for toxoplasma, rubella, CMV, HSV, and syphilis.

In all participants’ samples, the anti-ZIKV IgM antibodies were detected using the serological assay (MAC ELISA—CDC protocol) with Emergency Use Authorization (EUA) approval from the United States Food and Drug Administration (FDA) and performed in parallel to anti-DENV IgM. The MAC ELISA reagents were provided by both, Centers for Disease Control and Prevention (CDC) (Fort Collins, CO, EUA) and BEI Resources (USA) (10). The EUA-approved molecular assay (TRIOPLEX rRT-PCR), USA (http://www.cdc.gov/zika/state-labs/index.html, accessed on 27 March 2021) was performed for the detection of ZIKV RNA, DENV, and CHIKV RNA on participants of the CG (ZIP study). Molecular assays (qRT-PCR) on other participant’s samples were performed as described elsewhere (4,5).

### 6.3. Follow-Up Assessments

After birth, the MERG has aimed to reassess the children of the MERG–PC at 3, 6, 12, 18, 24, 36, 48, and 60 months of age (Table 3). Children enrolled at any time point after birth complete the remaining assessments after their age of entry (i.e., a child who joined at approximately 8 months of age would have follow-up assessments at 12, 18, 24, 36, 48, and 60 months). The complete neurodevelopmental assessments with Bayley-III were performed at least twice during follow-up, but at different ages for different children.

The children of the CG were evaluated at 3, 6, and 12 months of age for a general physical exam; neurological assessment; and auditory, visual, and neurodevelopmental screening assessments [10]. Thereafter, they were followed according to the MERG–PC schedule.

## 7. Baseline Characteristics

Due to limited funding, loss of follow-up, and/or refusal to participate further in the study, it was not always possible for children to be assessed by all four of the core specialists teams at baseline (i.e., paediatrics, neurology, ophthalmology, and otorhinolaryngology). Children from MCCS and CG groups had their baseline assessment at birth, and children from OG and MERG–PWC groups, at the first evaluation by the MERG task force. Children from any of the four groups (OG, MCCS, MERG–PWC, CG) who had not completed at least one of the specialist assessments at any point during follow-up were excluded from the MERG–PC (see flowchart).

The baseline cohort included 711 children born between February 2015 and February 2019 (Figure 1). The median age at baseline for OG and MERG–PWC was 14.2 months (range: 0.7–43.7) (Table 4). Approximately 52% of the children in the cohort were female. The children’s characteristics at baseline, excluding CG, were the following (percentages were calculated based on observations without missing information): 32.6% (184/565) had microcephaly; 47% (263/559) had at least one physical abnormality; 29.5% (160/543) had at least one neurological abnormality; and 46.2% (257/556) had at least one ophthalmological abnormality. An assessment of microcephaly at birth was available for 462 of the children in the cohort, of whom 28.8% were diagnosed with microcephaly at birth. Children of CG were not included in this characterisation.

## 8. Key Findings and Publications

The preliminary results from the MCCS component of MERG–PC were the first to provide evidence linking microcephaly and in utero ZIKV infection [4]. The study found an overall matched odds ratio of 73·1 (95% CI 13·0–∞) for microcephaly and laboratory-confirmed ZIKV infection after adjustment. The final report of the case–control study [5] confirmed this conclusion and also provided evidence of the absence of associations between microcephaly and other proposed causal factors, such as exposure to the larvicide pyriproxyfen or receipt of vaccines during pregnancy.

More recently, data from the MERG–PWC were used to develop an algorithm for defining ZIKV infections during pregnancy using qRT-PCR, IgM, IgG3, and/or PRNT_50_ that integrates and utilises the full array of available ZIKV diagnostic tools [9]. This classification is useful as it helps researchers to make sense of discordant ZIKV qRT-PCR and serology results, which occur not infrequently in ZIKV observational studies. This algorithm was used to estimate the absolute risk of adverse outcomes in children according to the different degrees of evidence of exposure to ZIKV infection during pregnancy. Among the children with laboratory evidence of prenatal ZIKV exposure, the absolute risk of presenting with at least one adverse outcome compatible with CZS was approximately 20%; the risk of microcephaly was 3%; and the risk of combined outcomes, i.e., more than one outcome (microcephaly, imaging, neurologic, and ophthalmologic) was less approximately 1% [19].

This ongoing cohort has contributed to the understanding of the CZS spectrum with an initial description of a large series of 104 cases of children born with microcephaly in 2015 [20] and, subsequently, the description of EEG patterns and the treatment response in early epilepsy [21,22] and findings of paediatric neurodevelopment [23]. We also reported the frequency and characteristics of oropharyngeal dysphagia [18], cryptorchidism and its surgical findings [24,25], endocrine dysfunction [26], and adenoid hypertrophy in children with Zika-related microcephaly [27].

## 9. Strengths and Limitations

MERG formed and commenced its research studies in the midst of the microcephaly epidemic that emerged in Brazil in 2015. MERG–PC is the largest single cohort study of children with CZS. The participants in MERG–PC include the first neonates ever detected to have the CZS phenotype. The children have undergone a comprehensive array of baseline clinical, laboratory, and imaging assessments. Follow-up clinical assessments have been carried out by the same group of clinicians for all of the individuals. Laboratory diagnostics have been performed on the majority of participants. Testing for ZIKV was independent of the presence of CZS phenotypic features or microcephaly, which has facilitated investigations of individuals across the full spectrum of CZS phenotypes, from mild to severe.

The study protocols and questionnaires were shared across Brazilian states to enable harmonisation across the different studies investigating microcephaly and CZS. This process was key for ensuring a comprehensive clinical overview of the impact of congenital ZIKV infection. In addition, it provided the opportunity for the Zika Brazilian Cohorts Consortium to be formed, uniting most of the Zika virus clinical cohorts in Brazil [28].

MERG–PC also has limitations that mostly stem from the urgency of the MERG investigations, which commenced in the midst of the Zika and microcephaly epidemics. The recruitment strategy was complex and multi-faceted, which resulted in a heterogeneous study population with different ages at baseline. Due to finite funding and challenging social circumstances for the families recruited into the study, not all laboratory and clinical specialist investigation results are available for all of the study participants at all time points as specified in the study protocol. The emergence of COVID-19 in Brazil in the first half of 2020 also had significant impacts on MERG–PC, requiring the majority of face-to-face appointments to switch to telephone consultations. The ability to adapt to this style of working shows the resiliency of the professionals and families involved but also resulted in some limitations including the fact that children could not be physically examined directly by physicians during this period.

## 10. Where Can I Find Out More?

For more information about MERG and its projects, please see https://rede.tghn.org/collaborators/merg/, and https://zikaplan.tghn.org/zikaplan-at-work/congenital-zika-syndrome/ (both accessed on 27 March 2021)**.**

Due to the sensitive nature of the data and the potential for participant identification, MERG–PC data are not publicly available. Any researcher wanting to use MERG–PC data should contact MERG.

## Figures and Tables

**Table 1:** Recruitment of the children’s cohort.

Group	Recruitment Period	N of Children Eligible for Recruitment	N of Children Included in MERG_PC
Outpatient group (OG)	11/2015–04/2019	195	195
Microcephaly case–control study (MCCS)	01/2016–11/2016	273 (91 cases, 182 controls)	80 (34 cases, 46 controls)
MERG pregnant women cohort (MERG–PWC)	12/2015–06/2017	503	336
Control Group (CG)	03/2017–07/2018	-	100
MERG Paediatric cohort (MERG–PC)	10/2016–04/2019	971	711

**Table 2:** Baseline measurements in the MERG–PC.

	Population	Subgroup	Time Points	Tests
Questionnaires	Maternal	MCCS	After delivery	--
		MERG–PWC	At second study visit (at least 14 days following initial notification of rash)	--
		CG	During pregnancy and after delivery	--
	Paediatric	MERG–PWC	After delivery or at earliest possible time after joining the MERG–PC	--
Diagnostics	Maternal	MCCS	After delivery	ZIKV by qRT-PCR, IgM and/or PRNT_50_
				CHIKV, DENV, toxoplasmosis, and/or CMV by qRT-PCR, IgM, and/or IgG Rubella by IgM and/or IgG
		MERG–PWC	At first study visit (within 5 days of rash)	ZIKV by qRT-PCR, IgM, IgG3, and/or PRNT_50_
				DENV by PRNT_50_ Toxoplasmosis, rubella, and/or CMV in serum by IgM
			At second study visit (at least 14 days following initial notification of rash)	ZIKV by IgM, IgG3, and/or PRNT_50_
			At third study visit (after delivery)	ZIKV by IgM, IgG3, and/or PRNT_50_
		CG	Throughout pregnancy and after delivery	ZIKV by RT-PCR and IgM
			During pregnancy	Toxoplasmosis, rubella, CMV, HSV, CHIKV, DENV in serum by IgM and/or RT-PCR. Screening test for syphilis
	Paediatric	OG	At birth (evaluated by hospital, medical consultation, or MERG) or first assessment (evaluated by MERG)	ZIKV by qRT-PCR, IgM, and/or IgG (umbilical cord blood and/or CSF) Syphilis by VDRL TORCH by IgG and/or IgM CHIKV, DENV, toxoplasmosis, and/or CMB (umbilical cord blood by qRT-PCR) CHIKV and/or DENV by qRT- PCR (CSF) CMV in urine by qRT-PCR
		MCCS	After delivery	ZIKV in serum and CSF by qRT-PCR, IgM, and/or PRNT_50_
				DENV in serum by PRNT_50_ Toxoplasmosis, rubella, and/or CMV in serum by IgM
		CG	After delivery	ZIKV by RT-PCR and IgM (if indicated)
				Toxoplasmosis, rubella, CMV, HSV in serum by IgM. Screening test for syphilis
Clinical examinations	Paediatric	All groups	At first study visit, among specific subgroups, and/or upon indication	Imaging (CrUSS, CT)
			At first study visit	Paediatric clinical consultation
				Ophthalmologic assessment (RetCam, anatomic structural evaluation, visual acuity test)
				Otorhinolaryngologic assessment (including ABR screening)
				Neurologic assessment
Neurodevelopmental assessments	Paediatric	All groups	At 3-month assessment or at first visit if ≥3 months	SWYC (for CG: ASQ)
			At 6-month assessment or at first visit if ≥6 months	Bayley-III

TORCH: Toxoplasmosis, rubella, cytomegalovirus, herpes simplex virus. CrUSS: cranial ultrasound scan, CT: computed tomography, ABR: auditory brainstem response, SWYC: Survey of Well-Being of Young Children [17], ASQ: Ages and Stages Questionnaires.

**Table 3:** Follow-up assessments in the children’s cohort from 2016–present.

	Subgroup	Time Points	Tests
Clinical examinations	Children with microcephaly and/or other indication	24 months or as indicated	Imaging by MRI at 24 months EEG, CT, or MRI at any time during follow-up if medically indicated
	All groups	3 months 6 months 12 months 18 months 24 months 36 months 48 months 60 months	Paediatric clinical consultation
			Ophthalmologic assessment (Anatomic structural evaluation, visual acuity test)
			Otorhinolaryngologic assessment (including ABR screening)
			Neurologic assessment
Neurodevelopmental assessments	All groups	3 months 6 months 12 months 18 months 24 months 36 months 48 months 60 months	SWYC *
		At least twice within 36 months	Bayley-III

* SWYC: Survey of Well-Being of Young Children. Notes: (1) The timing of the assessments did not always correspond exactly with those stated above due to follow-up challenges described in detail in the limitations section; (2) children of the CG were evaluated by ASQ test instead of SWYC; (3) Bayley-III test was not routinely administered to children with microcephaly.

**Table 4:** Baseline cohort characteristics.

Characteristics	*n* or Median (Range)	%	
Age at Baseline (Months)			
Outpatient group (OG)	16.4 (0.9–43.7)	**Age group**	**n (%)**
		at birth	180 (25.3)
		>1 day to 6 months	97 (13.6)
		>6 to ≤12	114 (16)
		>12 to ≤18	170 (23.9)
		>18 to ≤24	62 (8.7)
MERG-PWC	11.6 (0.7–39.9)	>24 to ≤30	61 (8.6)
Microcephaly case–control study (MCCS)	- (at birth)	>30 to ≤36	22 (3.1)
Control Group (CG)	- (at birth)	>36	5 (0.7)
Sex *			
Female	317	51.9	
Male	294	48.1	
Microcephaly at first assessment *			
Yes	184	30.1	
No	381	62.4	
Not available	46	7.5	
At least one physical abnormality at first assessment *			
Yes	263	43	
No	296	48.5	
Missing	52	8.5	
At least one neurological abnormality at first assessment *			
Yes	160	26.2	
No	383	62.7	
Missing	68	11.1	
At least one ophthalmological abnormality at first assessment *			
Yes	257	42	
No	299	49	
Missing	55	9	

* This information refers to the following groups: OG, MCCS, and MERG–PWC.

## Data Availability

Data cannot be shared publicly because public availability would compromise patient privacy. De-identified data can be made available upon reasonable request from qualified investigators by contacting the Programa de Pós-Graduação em Ciências da Saúde (PPGCS) da Universidade de Pernambuco (UPE) at ppg.cienciasdasaude@upe.br.
